# Supplementary material for: Giant honeybees (Apis dorsata) trade off defensiveness against periodic mass flight activity
Source: PLoS One. 2024 Apr 17;19(4):e0298467. doi: 10.1371/journal.pone.0298467 (PMC11023302; doi:10.1371/journal.pone.0298467)
Supplement: S1 Text — (DOCX) [file pone.0298467.s001.docx]

Equation 1. Measurement of the framewise change of pixel luminance

| (1) | ∆ lum_px_ = lum_px_ (t_i_) - lum_px_ (t_i-1_) | The term *lum*_px_ defines the 24-bit RGB luminance (R: 0-255; G: 0-255; B: 0-255) of a single pixel area [*px*] in frame *f*_i_ at time *t*_i_. The differential luminance value ∆ *lum*_px_ describes the change in luminance of the same pixel area in the time interval ∆*t* between the two time points [*t*_i-1_, *t*_i_] corresponding with the interval between the two sequential frames [*f_i_*_-1_, *f*_i_]. Since we used a frame rate of 50 Hz, this time interval in which we measured the differential luminance (∆*lum*_px_) was 20 ms. |
| --- | --- | --- |

Equations 2. Definition of motion-active pixels

| (2a)  (2b) | _mot_a [px] = 1  _mot_a [px] = 0 | *Motion-active state*: a selected pixel area [*px*] with ∆ *lum_px_* > *lum*_th_  *Motionless state:* for a selected pixel area [*px*] with ∆ *lum_px_* <= *lum_th_*  The RGB luminance values (∆*lum_px_*) form the basis for the distinction between motion-active and motionless state, which is determined for each individual pixel area [*px*]. Motion activity was detected at this pixel area (Eq#2a) when the RGB luminance exceeded the threshold value *lum_th_*= 10. For the 24-bit RGB luminance with the range of 0…255 for each of the three RGB domains this threshold results in *lum_th_* [R] * *lum_th_* [G]* *lum_th_* [B] = 1 000, which corresponds to the share of 6.03086 E-05 in the total data space. |
| --- | --- | --- |

Equations 3. Measurement of the framewise change of pixel luminance

| (3a) | _mot_A [mfa, sz_j_, t_i_] = $\sum_{k=1}^{k= 10000}$(_mot_a_px_ [k] ) | With *_mot_A*, motion activity; *mfa*, episode of the MFA activity; *sz_j_*, the selected surveillance zone (*j* =  1-5) with the size of the square of 100 *px* X 100 *px* = 10 000 [*px*] in the originally recorded frame; a single pixel is denoted here as *px* concerning a length or as [*px*] concerning an area; *_mot_a_px_*[*i*], the value assigned in Eq#3a to the area [*px*] which is equivalent to the number *k* of the respective pixels confirming motion activity (*_mot_a* [*px*] = 1) or motionlessness (*_mot_a*  [*px*] = 0) as defined in Eq#2. For each frame *f*_i_ at the timepoint *t_i_*, the motion activity *_mot_A* is quantified for each surveillance zone *sz_j_* by summing up the motion-active pixels [*px*] contained therein. |
| --- | --- | --- |
| (3b) | rel _mot_S [mfa, sz_k_] = $\sum_{j=fstart}^{j= fend}$(_mot_A [j] ) / n_ff_ [sess_exp_] | *f_start_* , *f_end_* are the frames at the starting and ending time *t_start_*, *t_end_*  of the respective experimental session *sess_exp_*; *n_ff_* [*sess_ex_*_p_] is the number of frames of the respective session. Therefore, *rel _mot_S* (Eq#3b) is the relative expression of the sum of motion activity per frame throughout an entire experimental session (*sess_exp_*). This normalization takes into account that the movement activity in the experimental sessions can only be compared if the periods of different lengths are taken into account. |
| (3c)  (3d) | max rel _mot_S =  =MAX (rel _mot_S [mfa, sz_1-5_, sess_exp_ = 1.. max_sess_])  rel_2_ _mot_S [mfa, sz_k_, sess_exp_] =  =rel _mot_S [mfa, sz_k_, sess_exp_ ] / max rel _mot_S | From all experimental sessions (*sess_exp_*), the total maximum of the values *rel _mot_S* (Eq#3c) is determined from all surveillance zones (*sz*_1-5_).  Then the value *rel_2_ _mot_S* is calculated by its normalisation with the total maximum (Eq#3d) and thus provides the relative image-based motion activity per experimental session in one of the five surveillance zones (*sz*_k_). between the limits of 0 and 1. |

Equations 4. Calculation of the proportion of frequency components for collective motion pulses

| (4a) | Δ _mot_A [t_k_, Δt_Δff_] = _mot_A [t_k_] - _mot_A [t_k_ +Δt_Δff_] | This algorithm is a substitution of the Fourier transform, which decomposes functions into their frequency components.  In a first step, for the given session nP_d_ 3 (*mfa*_1_), according to the definition of *_mot_A* [*mfa*, *sz_j_*, *t_k_*] (Eq#3a), the difference of the values of the motion activity (Δ*_mot_A* [*t_k_, Δt_Δff_*]) between two time points [*t_k_*] and [t*_k_* +Δ*t*_Δ_*_ff_*] was determined sequentially, i.e. from the beginning to the end of the selected session (Eq#4a). These Δ*_mot_A* values were sorted into *i_cat_* = 25 logarithmically scaled categories from the minimum Δ*_mot_A* value at the negative side to Δ*_mot_A* = 0, and then also into further 25 categories on the positive side from the value Δ*_mot_A* = 0 to the maximum Δ*_mot_A* value. In the next step, one of the two difference ranges is used for the following evaluation steps, since only the upward or the downward part of the motion activities is needed for the characterization of the slow wave to be analyzed. |
| --- | --- | --- |
| (4b)  (4c)  (4d)  (4e) | wN_c_ [i_cat_, Δt_Δff_] = N_c_ [i_cat_, [Δt_Δff_] * UL [i_cat_, [Δt_Δff_]  SUM wN_c_ [Δt_Δff_ ] = $\sum_{icat=1}^{icat=25}$wN_c_ [i_cat_, [Δt_Δff_]    max SUM wN_c_ = MAX (SUM wN_c_ [Δt_Δff_ ])  rel SUM wN_c_ [Δt_Δff_ , sz_1-5_] =  = SUM wN_c_ [Δt_Δff_ , sz_1-5_] / max SUM wN_c_ | The number of cases *N_c_* [*i_cat_*, Δ*t*_Δ_*_ff_*] (Eq#4b) which was found in the motion category *i_cat_* were weighted to the value of Δ*_mot_A* at the upper limit of the respective category *UL* [*i_cat_*]. This was done for each of the selected time spans Δ*t*_Δ_*_ff_*.  In a next step, the sum of all cases weighted across the 25 categories (Eq#4c) was determined on the positive side of the spectrum. This is repeated for all time periods Δt_Δff_ selected for the assessment of the differential values of motion strength Δ*_mot_A* [*t_k_,* Δ*t*_Δ_*_ff_*].  From all *SUM wN_c_* values (Eq#4c), the maximum value was determined (Eq#4d) in order to scale these values comparably between 0 and 1 as far as the nest areas *sz*_1-5_ are concerned (Eq#4e). These *rel SUM wN_c_* values (ordinate) are then plotted against the frequency scale (abscissa) in Fig. 6A,B. |

Equations 5. Assessment of the number of cases per category of motion activity forming the histogram-based motion spectrum

| (5a) | Increment_50_ =  = (LOG_10_ (max _mot_A [mfa] - min _mot_A [mfa])) / 50 | The value *Increment_50_* (Eq#5a) is the fiftieth part of the logarithmic value of the maximum value of motion activity max *_mot_A*. The min *_mot_A* [*mfa*] value of the distribution of motion activity values of the selected episode *mfa* is set in the paper mathematically as 0 [*px*], equal to zero motion activity (defined as zero motion-active pixel per frame according to Eq#3a) in the course of more than 60 000 frames evaluated for each episode. |
| --- | --- | --- |
| (5b)  (5c)  (5d)  (5e) | _LL_LOG_mot_ [I_h_] =  = LOG_10_ (min _mot_A [mfa]) + Increment_50_ * (I_h_-1)  _UL_LOG_mot_ [I_h_] = _LL_LOG_mot_ [I_h_] + Increment_50_  _LL_LOG_mot_ [I_h_] < LOG_10_ (_mot_A [f_i_, mfa]) < _UL_LOG [I_h_]  rel_1_ _mot_A [I_h_] = n_ff_ [I_h_] / N_ff_ [sess_exp_] | Therefore, in any case the general lower limit [*LL*] of the entire distribution of the motion activity of the selected episode is set in the paper mathematically as 0 [*px*], (equal to zero motion activity) per surveillance zone (*sz*_1-5_) while the general upper limit [*UL*] of the range of motion strength is assessed is the maximal value of motion activity (according to Eq#3a) determined in all five surveillance zones [*sz*_1-5_] per *mfa* episode (Eq#5a). The respective lower and upper limits of the 50 histogram intervals *I_h_* are calculated according to the Eq#5bc.  A single case of motion event *_mot_A* (Eq#3a) at the time *t_i_* concerning the captured image *f_i_* refers to the number of motion-active pixels per surveillance zone and this value is then as its logarithmic value LOG_10_ (*_mot_A*) sorted according to the Eq#5c,d into the motion strength category *I_h_*.  For each histogram interval *I_h_*, this sorting process yields a value indicating the number of cases concerning this motion strength interval (*n_ff_* [*I_h_*]), each of these cases referring to a single image. This number must be normalized to the length of the corresponding experimental session for comparability with other sessions (Eq#5e). This length of session is quantified with the number of frames involved *N_ff_.* The result *rel_1_ _mot_A* presents itself as a value for the motion strength in a relative term. |
| (5f)  (5g) | max rel_1_ _mot_A =  = MAX (rel_1_ _mot_A [I_h_ = 1 .. 50; sess_exp_ = 1 .. max_session; sz_1-5_])  rel_2_ _mot_A [I_h_] = rel_1_ _mot_A [I_h_] / max rel_1_ _mot_A | Lastly the maximum *rel_1_ _mot_A* value (Eq#5f) was assessed regarding all experimental sessions (e.g. *sess_exp_* = 1..17 for *mfa*_1_), and for all nest areas (*sz*_1-5_), but separately for the episodes *mfa*_1-3_ (see Fig. 4 A-C). This value was used to normalize the *rel_1_ _mot_A* data (Eq#5e) to range between 0 and 1 (*rel_2_ _mot_A* [*I_h_*]: Eq#5g) for easier comparison. |

Equations 6. Assessment of the size of the tail areas of the histogram-based motion spectrum

| (6a)  (6b) | n_i_ = rel_2_ _mot_A [I_h_ = i]  n_k_ = MAX (n_i_) (for n_1_ , ..., n_k_, ..., n_50_) | In each histogram-based motion spectrum created with Eq#5g, the interval *k* was determined which has the maximum of the cases (Eq#6a,b). This is repeated for all the motion spectra of the entire series of sessions (*j* = 1 to *max sess_exp_*) of each episode (*mfa*_1-3_) selected, and separately for all five surveillance zones (*sz_1-5_*). |
| --- | --- | --- |
| (6c) | k [P_d_] _j_ = (k [nP_d_] _j-1_ + k [nP_d_] _j+1_) / 2 | However, because the P_d_ sessions in the semi-quiescent state have clearly bimodal distributions (Fig. 7A), the referenced maximum of the motion profile for a given [P_d_]*_j_* session was determined here in a pragmatic way, by the mean of those positions where the two nP_d_ sessions before and after this P_d_ session ([nP_d_] *_j-_*_1_, [nP_d_] *_j_*_+1_; Eq#6c) had their maxima in their motion spectra. |
| (6d)  (6e)  (6f) | UL = k + 5; LL = k - 5  uTa = $\sum_{i=UL}^{50}$(rel_2_ motA [i] )  lTa = $\sum_{i=1}^{i=LL}$(rel2 motA [i] ) | On both sides of the maximum in the motion spectrum (at the position *I_h_* = *k*), the tails are set with the respective distance of five intervals of the motion strength (where the interval *I_h_* is defined according to Equ#5). Thus the left tail of the spectrum contains the *rel_2_ _mot_A* [*I_h_*] values (Eq#5g) in the interval range [*I_h_* = 1 ... *LL*], and the right tail in the interval range [*I_h_* = *UL* ... 50] (Eq#6d-f). The characteristic values of both tail ranges (u: upper range; l: lower range of the spectrum) are then determined by the respective sums according to Eq#6e,f. |
| (6g) | q_s_ = uT_a_ / lT_a_ | Finally, the index *q_s_* (Eq#6g) is used as a measure of skewness of the motion spectrum. This relation does not require normalization, but on the other hand it is also independent of the position of the distribution on the spectral scale of motion strength. |

Equations 7. How to calculate the base motion activity during mass flight for the comparison of episode *mfa_3_* with episodes *mfa_1,2_.*

| (7a)  (7b) | Δ _mot_A _mf_ [I_h_ , nP_d_] = _UL_LOG _mot_A [I_h_, nP_d_] - _LL_LOG _mot_A [I_h_, nP_d_] (see Eq#5c-e)  V_mf_ [I_h_] = rel_2_ _mot_A [I_h_] * (Δ _mot_A [I_h_] )^10  see Eq#5g for rel_2_ _mot_A [I_h_] | The logarithm of the motion activity (Eq#3) is calculated from the upper limit and the lower limit of an histogram interval of a nP_d_ session. Subsequently, these class measures are de-logarithmized (Eq#7b) to form the products of the pairs of motion magnitudes and the relative class-specific number of cases, and thus, all partial products are summed up (Eq#7c). |
| --- | --- | --- |
| (7c)  (7d) | rel _mot_S _mf_ [mfa, sz_i_, sess_exp_] = $\sum_{Ih=1}^{Ih = 50}$ V_mf_ [I_h_] [sz_i_, sess_exp_]  Q _mf_ [nP_d ,_ mfa_3 versus 1_ ] =  rel _mot_S _mf_ [mfa_3_, sz_i_, sess_exp_] / rel _mot_S [mfa_1_, sz_i_, sess_exp_]  for all nP_d_ sessions | If one forms the quotient (Eq#7d) between the two cases over *mfa*_1_ and *mfa*_3_ (Eq#7c) and takes the rel fraction of the episode *mfa*_1_ in the denominator (Eq#7d), the rel fraction *Q _mf_* [nP_d ,_ *mfa*_3 versus 1_ ] with respect to the basic motion activity under nP_d_ conditions in MFA is obtained for the episode *mfa*_3_. |

8. Matching the empirical skewness data with two motion inhibition models.

| (8a)  (8b)  (8c) | Y [0.6 min] = 1  Y [0.972 min, ., 10.7 min] = 1, ..., 0  Y [11 min, .,] = 1 | The hypothesis of *progressive-inhibition-during-mass-flight* (*pim*) hypothesis assumes that defensiveness is progressively inhibited during MFA and that this inhibition is then lifted again in a rapid change.  Therefore, in this model, the Y-value, corresponding to the skewness value in the empirical data [Eq#6g], was defined for the episode *mfa_1_* at the time 0.6 min with the value 1, from minute 0.972 to min 10.7 this Y-value drops linearly from 1 to 0, at minute 11 it is set again to the value 1, and until the end of the experiment this value remained at 1 [Eq#8a-c]. |
| --- | --- | --- |
| (8d)  (8e)  (8f)  (8g)  (8h) | Y [0.6 min] = 1  Y [0.972 min, ., 10.7 min] = 1, ..., 0  X = Radius * cos (α ), Y = Radius * sin (α )  Radius = 1  Y [11 min, .,] = 1 | The hypothesis of *inhibition-proportional-to-mass flight* (*ipm*) states that the ability to defend is reduced proportionally to the amplitude of the motion turbulence occurring during the MFA.  In this case, the model was defined for the episode mfa_1_ roughly along a circular shape, at min 0.6 with a value of 1, from min 0.972 – min 10.7 according to the circular [Eq#8f].  Here, the radius was defined with the value 1, and the value alpha ranged from 180 to 360°, thus representing a semicircular depression related to the time duration of the actual episode *mfa_1_*, corresponding to the time points from min 0.6 to min 10.7.  The Y-values of this model were set back to 1 for the further time axis from minute 11 onwards. |
| (8i)  (8j) | Rel q_s_ (sz_i_) = 0 … 1  PCC = Match Rel q_s_ (t [P_d_] _i_) ‖ Y (t [P_d_] _i_) | The empirical data, the *q_s_* time profile of P_d_ phases [Eq#6g] per surveillance zone were normalized between 1 and 0.  For each of the time points of the empirical data, the relevant y-values of the model were interpolated, the squared distances between the empirical values and the models were calculated. The relativised sum of the squared distances can thus be used as a measure of agreement between model and empirical data. In this paper, however, the Pearson correction coefficient was preferred as a measure of agreement between model and empirical data [Eq#8j]. |

Equations 9. Definition of trade-off parameters

| (9a) | y = A / x (with A = 1) | In a tradeoff, two variables are in an antagonistic relationship that can be represented mathematically as a hyperbolic function (Eq#9a), where the parameter A is responsible for spreading the curve away from the zero point [0,0] and has been defined as a value of 1 in the present tradeoff models. In the normalised form, the data then also lie between 0 and 1 and can be plotted as variables x,y on a linear scale. |
| --- | --- | --- |
| (9b) | log x + log y = 1 | Double logarithmically scaled, the data can be used as a reference with the linearly transformed hyperbolic function [Eq#9b], which extends as a diagonal from [0,1] to [1,0] at an angle of -45°. |

Equations 10. Definition of trade-off parameters with histogram-based spectra

| (10a) | State of Defensiveness  q_s_: q_s_ >1; q_s_ = 0; q_s_ >1 | The parameter *q_s_* [Eq#7g] was chosen to describe the apparent defensives. This describes the skewness of the histogram-based spectrum: If *q_s_* > 1, the spectrum becomes bimodal or at least has residual bimodality, i.e. shimmering waves are depicted in the spectrum. A value of *q_s_* = 1 or slightly below is the normal monomodal state of the quiescence. If this value drops further, it means that the motion activity in the selected collective of the surveillance zone is inhibited by external stimuli. |
| --- | --- | --- |
| (10b) | State of MFA:  k [P_d_] _j_ = (k [nP_d_] _j-1_ + k [nP_d_] _j+1_) / 2 | For a given experimental session, the size class *k* at which the maximum number of frames occurred was determined in a histogram-based spectrum. For the description of the current excitation level for a P_d_ session of the colony, the mean values of the corresponding size classes of the respective preceding and subsequent nP_d_ sessions were taken, both for nMFA and MFA.  Both parameters were normalized between 0 and 1. The normalization is given by the reference to the maximal value of defensiveness [Eq#10a] and MFA value [Eq#10b] in the surveillance zone per episode. |

Equations 11. Assessment of quantile-based motion spectra

| (11a)  (11b) | LL [q_i_] = 1 / n_q_ * (i-1) and  UL [q_i_] = 1 / n_q_ * i with i = 1 to n_q_  LL_w_ [q_i_] = 1 / n_q_ * (i-1) * max _mot_A and  UL_w_ [q_i_] = 1 / n_q_ * i * max _mot_A | *Definition* *of the abscissa for a weighted quantile*-*based spectrum range.* Given a choice of *n_q_* = 40 quantiles and a quantile range of 0 - 1 (that is: 0% to 100%, in steps of 2.5%, scaled therefore in terms of percentiles) of the data, the lower (*LL* [*q_i_*]) and upper (*UL* [*q_i_*]) limits of a quantile [*q_i_*] are defined with Eq#11a. To explain, the range of the first quantile is from *min _mot_A* to *_mot_A* [*UL_q_*_=1_], and the last quantile is from *_mot_A* [*LL_q=40_*] to *max _mot_A*, with *_mot_A* values defined according to Eq#3a. In a next step, the quantile ranges (abscissa values of the quantiles) were weighted (*LL_w_*, *UL_w_* ) with the maximum *_mot_A* value found in the respective sessions of the corresponding episode [*mfa*_1-3_] (Eq#11b). |
| --- | --- | --- |
| (11c) | Δ _mot_A [q_i_] = _mot_A (UL_w_ [q_i_]) - _mot_A (LL_w_ [q_i_]  with LL_w_ [q_i_] = UL_w_ [q_i-1_] | *Scaling of the ordinate of the quantile-based motion spectrum.* The motion value that characterizes the motion activity at the quantile position *q_i_* is determined by the difference between the quantile values at the upper and lower limits of the quantile interval (Eq#11abc). The motion values of the following four arousal conditions are summarized:  (1) [nP_d_, nonMFA] the state of semi-quiescence without excitation by the dummy presentation;  (2) [P_d_, nonMFA] the state of semi-quiescence with dummy presentation;  (3) [nP_d_, MFA] the state of mass flight without excitation by the dummy presentation;  (4) [P_d_, MFA] the state of mass flight with dummy presentation.  This kind of summarization is explained by the example of the semi-quiescent condition without dummy [nP_d_, nMFA, *sz_5_*, *mfa*_1_]: The maximum value of the motion activity was in the surveillance zone sz_5_  with *max _mot_A* = 1167.5833 px; the respective number of cases (= frames) was N_ff_ = 26 114 and comprised the selection of the following nP_d_ sessions: *sess_exp_* = 1,3,11,13,15 &17 (for schedules of arousal, see Fig. 4A_6_). The abscissa value was scaled with this *max _mot_A* value (Eq#11ab); as an example, the first quantile ranged therefore, from 0 px to 0.025 * max _mot_A = 29.1896 [*px*]; the lower value of this first quantile [*q_1_* ] was assessed as *min _mot_A* = 0.2222 [*px*], and its upper value with *_mot_A* = 4.5557 [*px*]. Therefore, the differential value *Δ_mot_A* [*q_2_*] (which belongs to the second quantile *q_2_*) was calculated as 4.5557 [*px*]– 0.2222 [*px*] = 4.3335 [*px*]. |

Equations 12. Calculation of the mean slope of the quantile-based spectrum in the selected part from 0.30 to 0.90

| (12a) | y = k_q_ x + d | Principally, the variables x,y are defined for this kind of spectrum as: y = LOG_10_ *_mot_A* [*q_i_*], and *x* = *q_i_*; for *i* = 0.01 to 0.99 (100 percentiles).  A second-degree regression polynomial is calculated from the spectral data in the upper range of the spectrum, from percentile *q_i_* = 0.30 to percentile 0.90. The slope value *k_q_* (Eq#11a) refers to data plotted as LOG_10_ *_mot_*A values (Eq#3) on the ordinate at the percentile position *x* = *q_i_* on the abscissa. These slope values were calculated in the same way for all experimental sessions (e.g. for the episode *mfa*_1_: *sess_exp_* = 1 to 17) and for all five surveillance zones (*sz_1-5_*). |
| --- | --- | --- |
| (12b)  (12c) | max k_q_ = MAX (k_q_ [sess_exp_ = 1 ..17, sz_1-5_]  rel k_q_ = k_q_ / max k_q_ | In a next step, the maximum inclination value [*max k_q_*] was determined from all experimental sessions and surveillance zones (Eq#12b). It is used to normalize the slope values to the range between 0 and 1 (Eq#12c). Negative slopes typically occur in the lower quantile range of the catenary curves, but this was not considered in this analysis due to the selection of the higher-order quantiles. In addition, mean values and mean errors were calculated for each experimental session with n = 5 surveillance zones. |

Equations 13. Calculation of the low base of distribution in quantile-based spectra

| (13a)  (13b) | Δ _mot_A [q_i_] = _mot_A (UL_w_ [q_i_] ) – _mot_A (LL_w_ [q_i_])  with LL_w_ [q_i_] = UL_w_ [q_i-1_]  For all q_i_ for I = 1 to 40 according to Eq#11c  _low_B = Means of quantile 1-10 = $\sum_{i=1}^{10}$ Δ _mot_A [q_i_] / 10 | In this case, the differences between the percentile values that occur in the comparison of two consecutive experimental sessions (*sess_exp_* -1, *sess_exp_*) are analysed.  For the calculation of the lower distribution basis of the difference values of the quantiles Δ *_mot_A* [*q_i_*]] of a certain experimental run, e.g. nP_d_ 1 (*mfa_1_*), quantiles of the levels 0.01 to 0.99 are again calculated from the difference values of the present motion spectrum (Eq#13a). The first 10 quantile values of the *Δ _mot_A* [*q_i_*] were averaged (Eq#13b) and provide a useful estimate of the basal value of the catenary curve _low_B of the selected session e.g. of nP_d_ 1 (*mfa*_1_). This value (Equ#13b) actually represents the middle of the respective working range where the quantile-dependent data density reaches its maximum in each experimental condition. |
| --- | --- | --- |
