# Supplementary material for: Giant honeybees (Apis dorsata) trade off defensiveness against periodic mass flight activity
Source: PLoS One. 2024 Apr 17;19(4):e0298467. doi: 10.1371/journal.pone.0298467 (PMC11023302; doi:10.1371/journal.pone.0298467)
Supplement: S2 Text — (DOCX) [file pone.0298467.s002.docx]

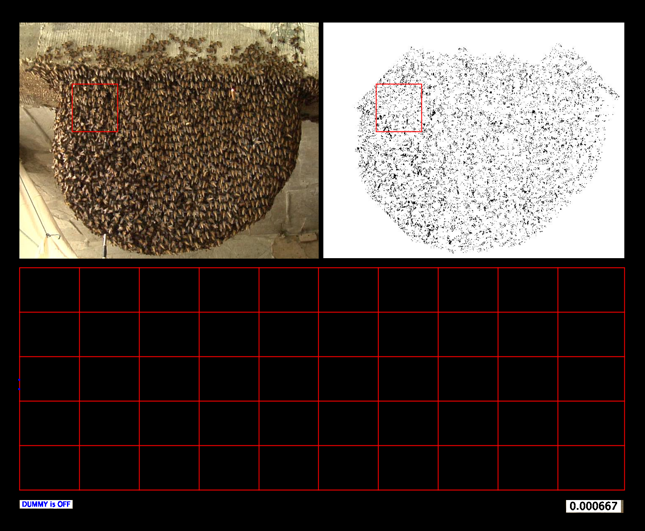

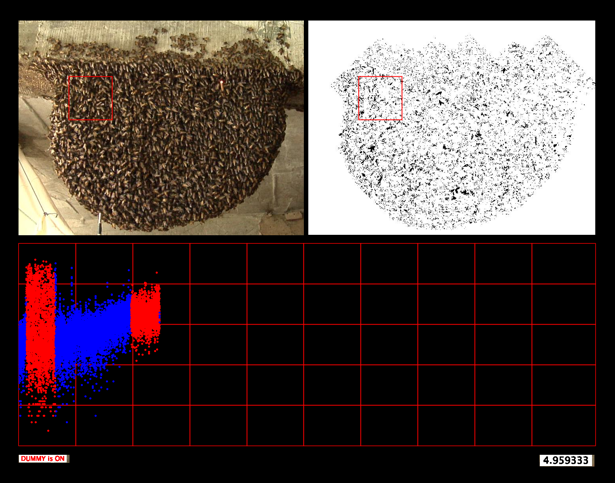


Supplementary movie 1.

Documentation of the MFA in episode *mfa_1_* from frame 1 (0.0007 min experimental time) to frame 14 876 (4.9470 min). The entire episode *mfa_1_*, shown here in four frame sequences, extends to a total of over 60 704 frames, covering 20.2373 min of film length; the frame rate is 50 Hz. Two types of experimental sessions alternate cyclically, namely those in which the experimental nest is observed undisturbed (without presentation of the dummy wasp: nP_d_ phase), with one in which a dummy wasp is presented (P_d_ phase). Such stimuli with dummy wasp were also continued during the mass flight episode. This film refers to four experimental sessions, from nP_d_ 1 to P_d_ 4 (see Fig 4A_6_) for the overview of the sessions). The MFA in this episode begins in session nP_d_ 3. The recording started a few minutes before the mass flight activity began, and was completed after 20.3 min, i.e. about 5 min after the MFA had stopped. Picture top left of each frame: video image of the nest; the camera (Panasonic HVX-200) recorded the nest from a little below the center of the nest. In the image sequence, the wasp dummy can be seen in the top left of the P_d_ sessions as it was pulled to and from the nest by computer control via a cableway in front of the nest at a constant speed. Right top image in each frame: Differential view of the upper left images with respect to the luminance differences between the two successive images (according to Eq #1a). The open red rectangles in both images represent the surveillance zone *sz_1_* to which the data in the lower graph refers. The point plot below shows the motion activity (according to Eq #3a) over the respective time of the recording. The time (abscissa: in minutes) of the respective image in the recording is indicated in the white rectangle at the bottom right. The blue dots refer to nP_d_ sessions, the red dots to P_d_ sessions. The motion activity is logarithmically scaled on the ordinate (for further information, see also Figs 2 and 4A). The following description and interpretation of the image sequence allows the observation of the motion behaviour at the nest in even greater detail.

| **Session**  (Fig 3A_1_) | **at Time**  in min | **Description** |
| --- | --- | --- |
| **nP_d_ 1** | 0.0000 | The film begins with a short session of nP_d_  1. In the mouth zone, many foragers are just dancing, e.g. follow them in the direction of 7 o'clock in the image; and peripheral to the mouth zone, at the right side of the image, probably as an unmistakable sign of incipient mass flight activity, some bees are wandering on the nest surface towards the center of the nest. On backtracking, it turns out that these bees came to the surface from inside the nest between the meshes of the bee curtain (and not via the mouth zone, which would be the normal way under nMFA). This observation was ultimately the reason for starting the experiment in its design with a sequence of nP_d_ and P_d_ phases. In this context, one can already see at this point in the nP_d_ 1 session that the blue point cloud (in the image in the lower recording area) is clearly rising at its upper envelope. This is a sign that the colony has entered mass flight mode. |
|  | 0.1640 | Under direction 4 o'clock in the image, a bee dances outside the mouth zone in straight-line mode. This behavior can also be interpreted as an alarm sign that the nest is about to switch to mass flight. |
| **P_d_ 2** | 0.2673 | The P_d_ 2 session begins and the dummy presentation continuously triggers shimmering waves. |
|  | 0.5300 | The presentation of the dummy wasp triggers strong shimmering waves. Nevertheless, the forager bees in the mouth zone continue their dances, although the shimmering waves pass over them with full force. |
| **nP_d_ 3** | 1.3133 | nP_d_ 3 session has started. General motion activity has increased in the mouth zone, some smaller shimmering waves are produced by a bee scanning in front of the nest at 1.876 min towards 11 o'clock. Migratory behavior on the nest surface in the right side periphery towards the center of the nest increases; foraging flights and waggle dances continue normally in the mouth zone (in the direction 7 o'clock). |
|  | 2.2400 | From this point onwards, the envelope curves at the upper and lower edges of the blue point cloud rise evenly and slowly, which is also expressed by the corresponding increase in the wandering rate of the bees in the nest periphery. |
|  | 2.9900 | Some bees are already landing in the direction at 12 o'clock in the upper middle of the nest, which indicates that some bees have already finished their defecation flight. Nevertheless, the restlessness is continuously increasing, that is, the mass flight mode continues to intensify. |
|  | 3.1700 | A bee lands in the direction at 1 o'clock in the upper nest zone. |
|  | 3.3900 | The general excitement increases, the blue dot cloud rises even more. (3.46 min) A bee flies off the upper nest edge at 12 o'clock direction in the image. |
|  | 3.6100 | The take-off rate of foragers from the mouth area is unchanged despite greatly increased mass flight activity. |
| **P_d_ 4** | 3.9400 | With the beginning of session P_d_4, the general agitation of the mass flight continues to increase strongly. |
|  | 3.9900 | Smaller shimmering waves are created, triggered by the wasp dummy, especially at the lower edge (in the direction at 6 o'clock), at the upper edge (in the direction 12-1 o'clock) and in the right periphery (in the direction 2-3 o'clock). In the surveillance zone *sz_1_* such waves do not appear, which is also shown by the red point cloud, which in this phase no longer shows any motion activity that goes beyond the envelope of the upper edge. However, the peak of mass flight activity has not yet been reached. |
|  | 4.5000 | Such shimmering waves are now occurring more and more sporadically in this P_d_ 4 session, and then only in the lower nest area (towards around 6 o'clock). In fact, we have arrived at mass flight mode, where scintillating activities are suppressed. |

Supplementary movie 2.


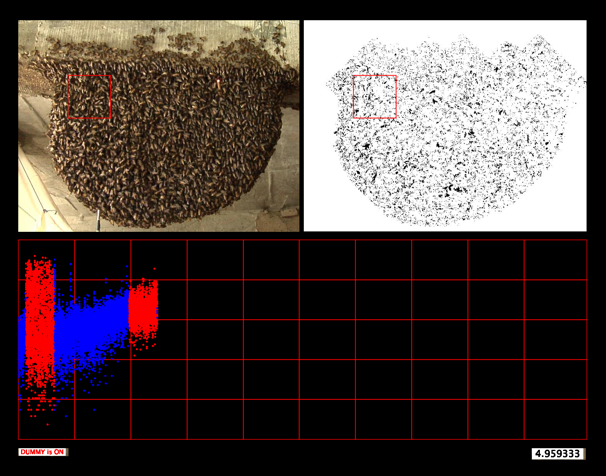

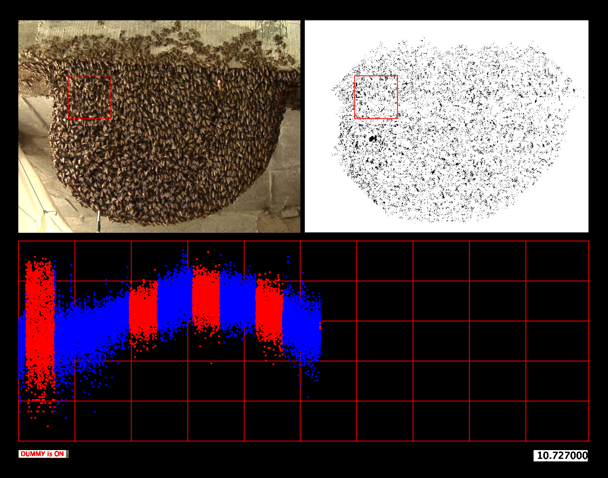


Documentation of the mass flight episode *mfa_1_* recorded from the surveillance zone *sz_1_* ranging from frame 14 876 (4.9470 min experimental time) to frame 32 177 frames (10.7270 min). In this image sequence, five experimental sessions are displayed from nP_d_ 5 to nP_d_ 9 (see Fig 4A_6_ for survey of sessions). This film reaches the peak in mass flight activity which is denoted by the soft peak of both envelope curves of the blue (nP_d_) and red (P_d_) point clouds. This film reaches the peak in MFA which is denoted by the soft peak of upper and lower envelope curves of the blue (nP_d_) and red (P_d_) point clouds. In total, the image sequence of episode *mfa_1_* ranges over 60 713 frames 20.2373 min) at a frame rate of 50 Hz, cyclically alternating between nP_d_ (without the presentation of the wasp dummy) and P_d_ (with the presentation of the wasp dummy) sessions which continued even during the MFA phase.

| **Session**  (Fig 3A_1_) | **at Time**  in min | **Description** |
| --- | --- | --- |
| **nP_d_ 5** | 5.0000 | There are now many bees flying around the nest, there is a real cloud of bees in the air between the camera and the nest. Most of the bees even take off from the periphery on the right side of the nest. It seems that the whole surface layer has detached from the bee curtain. In the mouth zone (towards 9 o'clock) you can still see some bee dancers doing their tail dance. |
|  | 5.6800 | Now it's really high noon as far as flying around is concerned. But in the mouth zone, the foragers that have just returned are still dancing. It seems that the behaviour at the nest in this mouth zone has remained unchanged, also in their basic movement activity of the surface layer there; there is not that apparent bustle as in all other regions of the nest. |
|  | 6.0603 | Some deep vertical furrows form on the surface of the nest, extending from the upper attachment zones to the lower edge of the nest. Are these furrows an increased detachment of the surface layer of the bee curtain? Or are they also a demarcation line separating the mouth zone from the rest of the nest? In any case, the envelope curves of the point cloud in surveillance zone *sz_1_* (plotted in the lower window in the monitor) show that the nP_d_ 5 session also sees the actual peak of movement activity during this mass flight. |
| **P_d_ 6** | 6.2060 | Start of the P_d_ 6 session. No shimmering activity can be observed on the nest surface. The periphery on the right side of the nest seems to calm down in terms of motion activity. The envelopes of the motion recording also decrease or at least remain at the same level. The rod illuminated by the Doppler vibrometer is exposed in double length due to the current removal of the surface layer. Waggle dancers are still visible in the mouth zone (around 8.30 o’clock in the image). There are outlier points below the lower envelope indicating that although the presentation of the dummy wasp has an effect on motion activity in MFA, it appears to be inhibitory in nature (see Fig 4A). |
|  | 6.7500 | Still no signs of shimmering on the nest; however, there are several furrows in the vertical direction in the bee curtain. One of them, just to the right of the mouth area, comes down to the lower edge. This furrow then also forms an indentation on the lower contour of the nest. The envelope curves of the motion recording signal decreasing motion activity. |
| **nP_d_ 7** | 7.3850 | The furrows fill up again. The hive in the upper part of the nest is also surrounded by bees again. The bees come back to the periphery and land there too. |
|  | 7.9750 | The measuring rod is covered less from the side again and the nest gets a beard at the lower edge where bees now land or fly away. |
|  | 8.3653 | The rod is free again. |
| **P_d_ 8** | 8.4837 | Session P_d_ 8 begins. Flight motions are significantly reduced. The increased motion activity registered in the monitor plot refers to the bees moving around the nest surface to find their target position in the nest. Still no tendency to shimmering discernible. |
|  | 8.8127 | Bees land rather than take off; the rod is still clear. There are some furrows on the nest, but more in the center than in the right periphery. On the far right of the image, the pattern of quiescent bees with their heads up and abdomens down is becoming clearer. |
|  | 9.0443 | Between this rightmost nest area and the still agitated area in the middle of the nest is a deepening furrow, which is also visible on the lower nest outline. The dipstick is still free. There is no shimmering activity. Instead, there are outliers below the lower envelope of the motion record, which appear to be typical of P_d_ conditions at this time of the MFA and where the dummy wasp exerts an inhibiting influence on motion activity. |
|  | 9.2976 | The boundary of the quieter region on the nest already extends to the vertical furrow in the nest that extends downwards from the illuminated measuring stick. |
|  | 9.3830 | Now the first signs of shimmering are appearing, albeit with a very weak expression, but typically where quiescence has already taken hold recognisably under the preceding nP_d_ conditions, namely on the right side of the nest. |
| **nP_d_ 9** | 9.4200 | Again, the nP_d_ 9 session begins after the presentation of the dummy wasp has ended. Landing bees on the far left of the nest trigger a faint flicker. This could also be the case in surveillance zone *sz_1_*, as some blue dots with higher movement intensity become visible in the monitor there. More and more bees are now landing in the upper nest areas as well. |
|  | 10.0700 | The motion pattern of the bees on the nest surface in the regions peripheral to the mouth zone clearly calms down and begins to differ from the more active regions of the mouth zone. Here, the waggle-dancing foragers seem to increase again in numbers. |
|  | 10.5176 | The measuring stick is increasingly covered by bees from the side; the movement activity envelopes (*sz_1_*) in the monitor come down from the higher movement intensities. Some vertical furrows on the surface remain, but only for short distances perpendicular to the nest. They apparently indicate that boundaries in the functional architecture of the nest still exist. |

Supplementary movie 3.


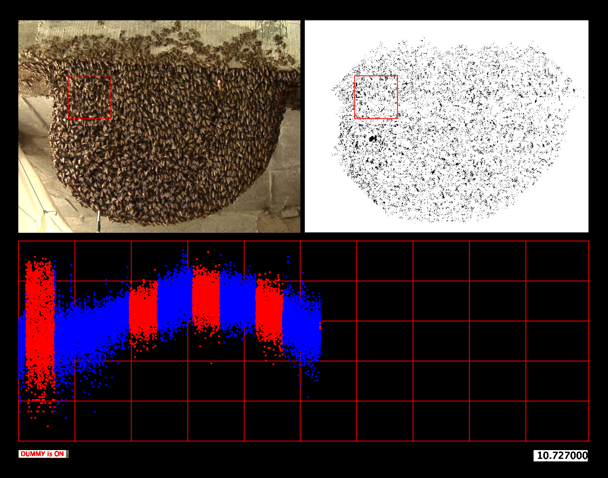

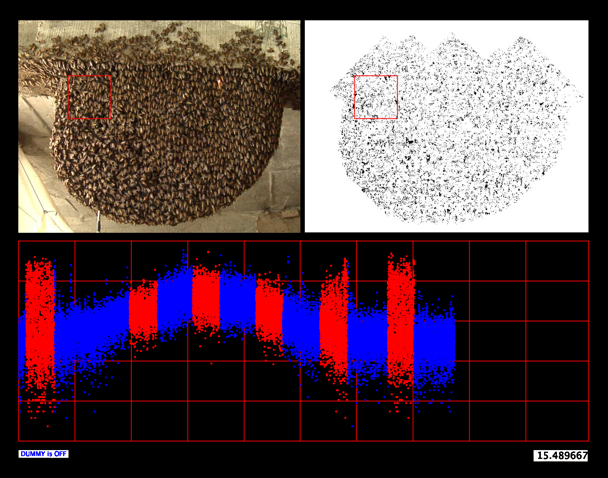


Documentation of mass flight episode *mfa_1_* from surveillance zone *sz_1_* from frame 32 177 (10.7270 min trial time) to frame 46 463 (15.4897 min). Four experimental sessions from P_d_ 10 to nP_d_ 13 are shown here (see Fig 4A_6_ for the overview of the sessions). This movie shows how the colony regains its full defense capability after the MFA has ended and the blue point envelopes of the nP_d_ plots in the monitor reach the status of quiescence. The red point clouds of the P_d_ sessions indicate active shimmering behavior through the upward motion peaks, but there are also some points below the extrapolated lower blue point envelopes in these phases, which also indicate cohorts inhibited by the dummy wasp presentation. In total, the image sequence of episode *mfa_1_* spans 60 704 frames (20.2373 min) at a frame rate of 50 Hz, cycling between nP_d_ (without presentation of the dummy wasp) and P_d_ (with presentation of the dummy wasp), which also continued during the MFA episode *mfa_1_*.

| **Session**  (Fig 3A_1_) | **at Time**  in min | **Description** |
| --- | --- | --- |
| **P_d_ 10** | 10.7540 | Experimental session P_d_ 10 begins, with the first stimulus conditions, smaller shimmering waves first appear in the 12 o'clock direction through 3 o'clock to 6 o'clock, but not in the mouth zone (in the 9 o'clock direction). |
|  | 11.0547 | In these regions (from the upper centre to the right side of the nest), the shimmering waves are obviously much more strongly expressed than in the red dot cloud on the monitor, which refers to the surveillance zone *sz_1_*, just above the mouth zone. |
|  | 11.3200 | The shimmering waves become stronger and reach all regions except the mouth zone (in the 8 o'clock direction). There, the returning foragers are active in the waggle dance. |
|  | 11.6020 | Now there are full-surface shimmering waves that cover the entire nest except for the mouth area. However, some smaller extensions of these waves come down from above the mouth area. |
| **nP_d_ 11** | 11.7940 | nP_d_ 11 starts now, with a late shimmering wave occurring after one bee landed up in the 12 o'clock direction and another flew over this region in a kind of search flight. Some nestmates land above in the 12 o'clock direction, causing small waves. |
|  | 12.1390 | Landing of returnees in the periphery has largely ceased, and wandering activity on the nest surface is also becoming less frequent. A vertical furrow appears vertically across the nest, a few bee widths from the center of the nest to the right, starting four bee lengths below the upper edge of the nest to the lower edge. In the contour line at the lower edge of the nest, the effect of this furrow is visible as a corner. |
|  | 12.3203 | The mouth zone expands towards 6 o'clock in the lower nest area, the forager bees that have returned home are already waggle-dancing there. |
|  | 12.5740 | The large vertical furrow is closed again, in the right periphery several bees are hanging in an oblique position, some of them are locomotively active. It seems that there are still some newcomers who have to find their target position with their new task to fulfil. |
|  | 12.9913 | Now most of the bees outside the mouth area are consistently in a vertical posture with the head up and the abdomen down, hanging almost without motion. The measuring bar in the upper nest area is again increasingly surrounded by the bees. |
| **P_d_ 12** | 13.1406 | The experimental session P_d_ 12 has started, shimmering on response of the dummy wasp presentation is observed in full strength. In the mouth zone (towards 8 o'clock), on the other hand, a wave arrives only rarely, sometimes an offshoot from above. In Jenny part, where apparently after the MFA the mouth zone has newly expanded (towards 6 o'clock), quite strong waves are generated. Obviously, there are two categories of mouth zone; in this one, at any rate, the bees at the nest surface are ready to take part in the defensive actions. |
|  | 13.5183 | Now, starting from the centre of the nest, really strong shimmering waves emerge, but (towards 8 o'clock) they still rarely arrive in the mouth zone. There, however, you can see some active tail dancers, which also do not participate in the vertieidation waves. |
| **nP_d_ 13** | 14.0643 | Sesssion nP_d_ 13 begins. Only remnants of shimmering waves appear nest-wide. |
|  | 14.2600 | A kind of flickering [57] is visible in the periphery on the right side towards the mouth area, no more wandering on the nest surface, but there are still some remnants of vertically oriented furrows in the bee curtain. |
|  | 14.3300 | A bee lands near the measuring rod at the top of the right side and triggers smaller shimmering waves. |
|  | 14.6113 | On the far right of the nest it has become really quiescent, in the upper center the bees are still moving in their mostly vertical posture. There is no real quiescence here yet. Several surface bees are not yet fully oriented in a vertical direction. |
|  | 14.7956 | There are waggle dances in the direction of 7 o'clock, so the mouth zone is also extended in its actual function in this direction below. |
|  | 15.3160 | A few bees are still moving around towards 12 o'clock on the nest surface, this is obviously still a reminiscence of the past MFA mode. |

Supplementary movie 4.


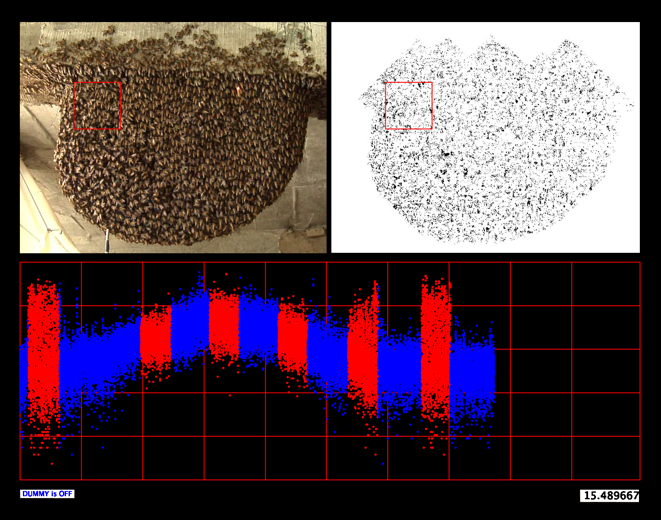

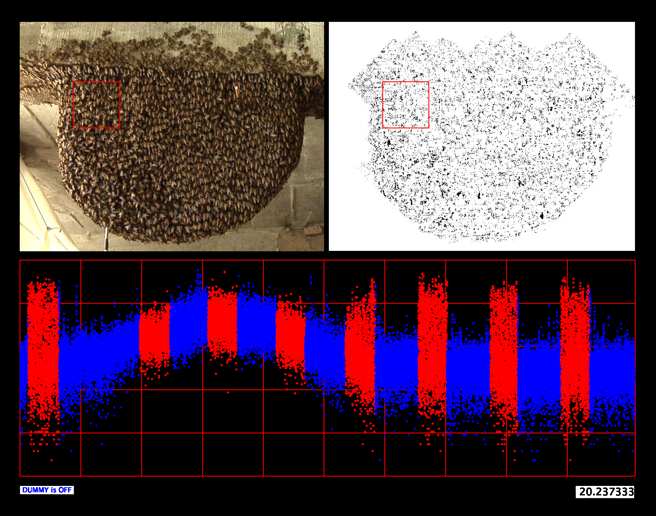


Documentation of mass flight episode *mfa_1_* from surveillance zone *sz_1_* from frame 46 463 (15.48967 min experimental time) to frame 60 704 (20.2373 min). Four experimental sessions from P_d_ 14 to nP_d_ 17 are shown here (see Fig 4A_6_ for the overview of the sessions). This film shows how the colony, which has already regained its full defensive capability after the MFA, continues to consolidate its functional nest structure. The envelopes of the blue nP_d_ sessions finally reached the quiescence level. The red dot clouds show upward motion peaks that can be attributed to shimmering events, and also downward motion outliers that indicate cohorts inhibited by the presentation of the dummy wasp. In total, the image sequence of episode *mfa_1_* spans 60 704 frames (20.2373 min) at a frame rate of 50 Hz, cycling between nP_d_ (without presentation of the dummy wasp) and P_d_ (with presentation of the dummy wasp), which was also continued during MFA.

| **Session**  (Fig 3A_1_) | **at Time**  in min | **Description** |
| --- | --- | --- |
| **P_d_ 14** | 15.5690 | The experimental session P_d_ 14 begins and shimmering waves spread powerfully over the entire nest. Obviously there are two qualities of regions in the mouth zone: on the one hand, the one monitored in the experiment by the *sz_4_* surveillance zone, and formed in the direction of 8-9 o'clock; and on the other hand, that zone which has only developed from this zone downwards to the right, in the direction of 6 o'clock. In the former zone there are only faint outcrops of shimmering activity, but these have been reflected in the time log as significant shimmering activity (see Fig 4A_4_,B_4_,C_4_). In the lower expansion arc of the mouth region, which also expands steadily over the course of a day, defensive capacities through shimmering are present from the beginning. |
|  | 15.7570 | Now, shimmering waves emanate from practically every point of the nest, but this depends on the respective, momentary position of the wasp dummy. It seems that most of the waves at this time emanate from the lower regions of the nest. |
|  | 16.0500 | A bee lands unusually in the middle of the nest. It is probably not a collecting bee, but one that had flown away late from the nest during the mass flight that had just ended. |
|  | 16.2203 | A dancer bee waggling over the nest in the direction of 12 o'clock, always straight ahead, without making a turn. |
|  | 16.3000 | Another bee lands in the middle of the nest, the bees in the upper nest region in the direction of 11-12 o'clock are now completely at quiescence again. The red dot clouds show full scale reactions with high motion amplitudes during shimmering, but also a drop below the base of the nP_d_ sessions, which is characteristic of shimmering sessions. |
| **nP_d_ 15** | 16.3733 | Session nP_d_ 15 begins. A deep furrow is on the left side of the measuring rod halfway down the nest. |
|  | 16.5543 | There are now no more furrows on the surface of the nest; this is also connected in time with the fact that the lower contour line of the nest has now also become rounder and that the angular character has dissolved. |
|  | 16.7766 | Forager bees are now increasingly leaving the mouth area from the 6-7 o'clock direction in the picture. The foraging operation is now set up intensively. |
|  | 17.3990 | It seems that the boundary of the mouth area moves a little to the left from the 6 o'clock direction to 7 o'clock. |
|  | 17.6576 | At 6 o'clock, a sharp dividing line runs vertically through the lower nest region: on its left side is the mouth zone with its lively movement activity, on the right side the bees on the bee curtain are quiescent. |
| **P_d_ 16** | 17.8180 | Session P_d_ 16 begins. Shimmering waves are initiated from the direction of 11 o'clock and 6 o'clock. |
|  | 18.4112 | Shimmering waves reach the mouth zone in the direction of 7 o'clock, but not 8 o'clock. |
|  | 18.7186 | A wasp scans over the nest and triggers more shimmering waves. |
| **nP_d_ 17** | 18.7600 | The last session of episode *mfa_1_* begins (nP_d_ 17). There are just a few small shimmering waves left, but they are all getting weaker and weaker. |
|  | 18.9786 | A bee scans and causes some faint shimmering waves. |
|  | 19.5966 | Flickering activity (in the sense of [57] can be observed in the upper central nest regions, but also intense tail dances in the mouth zone towards at 8 o'clock. |
|  | 19.7100 | Again a wasp flies over the nest, there are sporadic shimmering actions. |
|  | 19.8100 | A bee lands in the middle of the nest Holes are also created there, which may serve for ventilation and are visible as such in the normal video image; in the IR image they would appear colder [58]. |
