# Supplementary material for: Giant honeybees (Apis dorsata) trade off defensiveness against periodic mass flight activity
Source: PLoS One. 2024 Apr 17;19(4):e0298467. doi: 10.1371/journal.pone.0298467 (PMC11023302; doi:10.1371/journal.pone.0298467)
Supplement: S1 Table — (PDF) [file pone.0298467.s003.pdf]

Supplemental Table S1. Antibodies used for flow cytometry and immunohistochemistry.

Primary antibodies

Antigen	Antibody Description	Vendor	Catalog #
CD3e	Armenian hamster anti-mouse CD3e; clone 145-2C11	BioXCell	BP0001-1
CD4	Rat anti-mouse CD4; clone GK 1.5	Abcam	Ab133616
CD8	Rabbit anti-mouse CD8	Santa Cruz Biotechnology	sc-7188
CD11b	Rat anti-mouse CD11b; clone M1/70	BioXCell	BE0007
CD14	Rat anti-mouse CD14; clone M14-23	BioLegend	150102
CD18	Rat anti-mouse CD18; clone M18/2	BioXCell	BE0009
CD31	Rat anti-mouse CD31; clone MEC 13.3	BD Biosciences	553370
CD34	Rat anti-mouse CD34; clone RAM34	BD Biosciences	560238
CD105	Rat anti-mouse CD105; clone MJ7/18	Developmental Studies Hybridoma Bank	MJ7/18
CD117	Rat anti-mouse CD117; clone 180627	R&D Systems	MAB1356
CD133	Rat anti-mouse CD133; clone 13A4	Thermo Fisher Scientific	14-1331
CD146	Rat anti-mouse CD146; clone ME-9F1	BioLegend	134703
CD163	Rabbit anti-human/mouse CD163	Spring Bioscience	E18682
CD204	Rabbit anti-human/mouse CD204	Millipore Sigma	HPA000272
CD206	Goat anti-mouse CD206	Thermo Fisher Scientific	PA546994
CD209	Armenian hamster anti-mouse CD209b; clone 22D1	BioXCell	BE0220

Colec12	Goat anti-mouse Colec12	R&D Systems	AF3130
F4/80	Rat anti-mouse F4/80; clone F4/80	BioXCell	BE0206
GFP	Goat anti-GFP	R&D Systems	AF4240
Itga9	Goat anti-mouse Itga9	R&D Systems	AF3827
Ly6C	Rat anti-mouse Ly6C; clone Monts1	BioXCell	BE0203
Ly6G	Rat anti-mouse Ly6G; clone 1A8	BioLegend	127637
Lyve-1	Rabbit anti-mouse Lyve-1	AngioBio	11-034
Meca-32	Rat anti-mouse Meca-32; clone Meca-32	BioXCell	BE0200
MHC-II	Rat anti-mouse MHC-II; clone M5/114	BioXCell	BE0108
Nrp-1	Goat anti-mouse Nrp-1	R&D Systems	AF566
p67-pHox	Goat anti-human/mouse p67-pHox	Santa Cruz Biotechnology	sc-7663
Podxl	Goat anti-mouse Podxl	R&D Systems	AF1556
Pdpn	Syrian hamster anti-Pdpn; clone 8.1.1	BioXCell	BE0236
PD-L1	Rat anti-mouse PD-L1; clone 10F.9G2	BioXCell	BE0101
Sca-1	Rat anti-mouse Sca-1; clone Sca-1	ATCC	HB-215
SSEA4	Mouse anti-human/mouse SSEA4; clone MC-813-70	R&D Systems	MAB1435
Ter-119	Rat anti-mouse Ter-119; clone TER-119	BioXCell	BE0183
TLR4	Rabbit anti-mouse TLR4	Creative BioMart	Custom

Vegfr-1	Rabbit anti-mouse Vegfr-1	Santa Cruz Biotechnology	sc-316
Vegfr-2	Rat anti-mouse Vegfr-2; clone 89B3A5	BioLegend	121902
Vegfr-3	Goat anti-mouse Vegfr-3	R&D Systems	AF743

Secondary antibodies

Antibody Description	Vendor	Catalog #
Alexa fluor 488-goat anti-Armenian hamster IgG (H+L)	Jackson ImmunoResearch	127-545-160
Cy3-goat anti-Armenian hamster IgG (H+L)	Jackson ImmunoResearch	127-165-160
Alexa fluor 488-bovine anti-goat IgG (H+L)	Jackson ImmunoResearch	805-545-180
Cy3-F(ab)2-donkey anti-Goat IgG (H+L)	Jackson ImmunoResearch	705-166-147
Alexa fluor 488-donkey anti-mouse IgG (H+L)	Jackson ImmunoResearch	715-545-150
Alexa fluor 488-donkey anti-rabbit IgG (H+L)	Jackson ImmunoResearch	711-545-152
APC-F(ab)2 donkey anti-rabbit IgG (H+L)	Jackson ImmunoResearch	711-136-152
Alexa fluor 488-donkey anti-rat IgG (H+L)	Jackson ImmunoResearch	712-545-150
Cy3-donkey anti-rat IgG (H+L)	Jackson ImmunoResearch	712-165-150
Alexa fluor 647-donkey anti-rat IgG (H+L)	Jackson ImmunoResearch	712-605-150
Alexa fluor 488-goat anti-Syrian hamster IgG (H+L)	Jackson ImmunoResearch	107-545-142
Alexa fluor 647-goat anti-Syrian hamster IgG (H+L)	Jackson ImmunoResearch	107-605-142
