# Supplementary material for: Giant honeybees (Apis dorsata) trade off defensiveness against periodic mass flight activity
Source: PLoS One. 2024 Apr 17;19(4):e0298467. doi: 10.1371/journal.pone.0298467 (PMC11023302; doi:10.1371/journal.pone.0298467)
Supplement: Attachment — Submitted filename: rebuttal letter 2 to reviewer 1.docx [file pone.0298467.s009.docx]

Rebuttal letter

I would like to thank reviewer #1 for his close review of the revised manuscript, for his comments and queries, which were very helpful in significantly improving the manuscript during the final revision of the paper.

Gerald Kastberger

Reviewer #1: I thank the authors for their answers and revisions. The quality of the paper has increased immensely, it’s much more concise and a lot easier to read. I think the abstract could contain even less details about the methods used to reach the conclusions, but that may be a personal preference.

Below are a couple of details and typos that I noted:

L53: “In consequence the MFA intensity in mfa3 was significantly lower than in mfa1,2 which is an indication that the colony perceived external threats during MFA.” This sentence threw me off, I think because of the reversed relationship – I would rather argue that the MFA intensity is higher in mfa1,2 (than the baseline represented by mfa3).

L688-692 are repeated word for word L709-712. I think the 1st repeat was forgotten there?
L167: the lodge has a different name in the legend of Fig 1
L192: the “yellow” arrows are red.
L202-211: I’m not sure this section is necessary, and it seems to contradict previous statements (colonies need to be observed for 30min pre and post MFA according to this section, vs 10 min in the data presented).
L307: even … even
L473: <1Hz rather than 0.1, if I believe the x-axis of Fig 6?
L493-497: sentence repeated
L646-647: This sentence is weird, the grammar is wrong.
L652: bivouacs rather than bivaques
L658: the surface layer of the bee curtain
L704: even on the other side
L819: neither would be capable of shimmering. There and in other places, it is implied that the only defensive response available to the colony is shimmering. I think that is a bit of an over-generalization. If some bees can still detect the threat as suggested, they might still be able to individually fly out and sting/ball/harass the intruder, even if they have trouble building a strong collective response.
L842: first rather than lastly
L849: to an extent + to a certain degree

Jump from Fig 4 to Fig 6, Fig 5 not used?
Fig 7: red curves appear as dotted blue-red, making them hard to distinguish from the blue ones (and artboard visible in the back)
Just an idea - I feel like there could be a summary figure showing how both the MFA and defensiveness progress spatially across the nest (I mean a schematic representation, not another data-loaded figure) that would nicely wrap up the argument.

******************

# Query 1

L53: “In consequence the MFA intensity in mfa3 was significantly lower than in mfa1,2 which is an indication that the colony perceived external threats during MFA.” This sentence threw me off, I think because of the reversed relationship – I would rather argue that the MFA intensity is higher in mfa1,2 (than the baseline represented by mfa3).

# My answer:

No, this assumption is not correct. Figure 4 clearly shows that the blue dot plots of panels C are lower than those of panels A and B during MFA. This is a very basic result and therefore I explicitly mentioned it in the Abstract. Also note that the ordinates are the same for all three MFA episodes and refer to the original data without any normalisation, which are the logarithmised values of the number of moving active pixels per monitoring zone.

Nevertheless, for reasons of easier readiness we edited instead…

“*In consequence the MFA intensity in mfa3 was significantly lower than in mfa1,2 which is an indication that the colony perceived external threats during MFA.”*

.. the optional new version:

“*In contrast to mfa_1,2_, in mfa_3_ the experimental regime started with the cyclic presentation of the dummy wasp only after the MFA had subsided. As a result, the MFA intensity in mfa_3_ was significantly lower than in mfa_1,2_, suggesting that a colony is able to perceive external threats during the MFA.*”

******************

# Query 2 Repetition

Reviewer 1: L688-692 are repeated word for word L709-712. I think the 1st repeat was forgotten there?

# My comment:

Thank you for pointing this out. I have restructured both parts to avoid repetition.

Instead ...(Ad Questions to MFA on timing and restructuring of functional nest architecture☺

“*However, this also raises the question of whether a colony that is unable to develop defensive behavior is literally blind to external stimuli. Methodologically, this was investigated by exposing the experimental colony to a recurrent sequence of sessions (Fig 4) in which stimulation conditions in the form of presentation of a dummy wasp (P_d_) alternated with periods of no external threat (nP_d_). Size, proximity, and speed of this dummy (Fig 1) were adjusted to produce shimmering behavior under semi-quiescent conditions [36, 39, 41]. This strategy of experimental arousal was used here to determine and comparatively measure the willingness or ability of cohorts at the nest surface to develop defensive responses under conditions of sQu and MFA*.“

I apply …

“*However, this also raises the question of whether a colony that is unable to develop defensive behaviour is literally blind to external stimuli. Methodologically, this was investigated by exposing the experimental colony to a cyclically recurring sequence of external threats in the form of the presentation of a dummy wasp (Pd) (Fig. 4).”*

And instead...(Ad The three (a-c) recurrent excitation states of the giant honeybee nest under natural and experimental conditions:)

“*In a nest, the semi-quiescence state (a) prevails most of the day, which is more or less the "normal", undisturbed state of life of giant honey bee colonies (S1-S4 movies). In this state, except for the mouth zone, the bees hang in the bee curtain [16, 19, 20, 23] in several layers with the head up and the abdomen down. By external, visual or even mechanical stimuli, the state of shimmering is triggered on demand [9, 27, 36, 39, 40], which is manifested by a wavelike sequence of motion salvos on the nest surface, each involving hundreds of bees simultaneously or sequentially. To an outside observer, such undulating patterns appear like a visible alarm signal that has evolved primarily to deter predatory wasps [9, 40] that would otherwise approach nests unrestricted to strike prey. Furthermore, there is evidence [26, 27] that with this behaviour, the colony is able to inform the nest mates about the current threat situation, even on both sides of the central honeycomb*.”

*“(b) This state of semi-quiescence of the nest and its readiness for defensive responses such as shimmering is interrupted at regular intervals during normal daytime operation [42-45] by the state of the MFA.”*

*(c) Finally, for the present study, the experimental colony was additionally exposed to a recurrent sequence of external stimuli during the sQu and MFA phases, which were themselves autonomous and self-organised [16, 84, 85], alternating stimulation conditions in the form of the presentation of a dummy wasp (P_d_) with periods of no external threat (nP_d_). The size, proximity, and speed of this dummy (Fig 1) were adjusted to elicit shimmering behavior under sQu conditions [14, 23, 36, 39]. This type of external experimental stimulation was used here to comparatively measure the ability of cohorts at the nest surface to develop defensive responses also under MFA conditions.“*

We apply…

„*This state of semi-quiescence of the nest and its readiness for defensive responses such as shimmering is interrupted at regular intervals during normal daytime operation [42-45] by the state of the MFA (b).*

*For the present study, the experimental colony was additionally exposed to a recurrent sequence of external stimuli (c) during the sQu and MFA phases, which were themselves autonomous and self-organised [16, 84, 85], alternating stimulation conditions in the form of the presentation of a dummy wasp (P_d_) with periods of no external threat (nP_d_). The size, proximity, and speed of this dummy (Fig 1) were adjusted to elicit shimmering behavior under sQu conditions [14, 23, 36, 39]. This type of external experimental stimulation was used here to comparatively measure the ability of cohorts at the nest surface to develop defensive responses also under MFA conditions.* “

******************

# Query 3

L167: the lodge has a different name in the legend of Fig 1

# My comment:

Eden Jungle Resort is nowadays the correct name

Done

******************

# Query 4

L192: the “yellow” arrows are red.

# My comment:

Fig 3 was additionally edited!

Edited text:

(Fig 1B-C; see the red arrows in Fig 2A_2a_, and in Fig 3).

Done

******************

# Query 5

Reviewer 1: L202-211: I’m not sure this section is necessary, and it seems to contradict previous statements (colonies need to be observed for 30min pre and post MFA according to this section, vs 10 min in the data presented).

# My comment:

Instead…

„*In this paper we examine the question of how the collective defense changes during MFA of an Apis dorsata colony. To do this, it is necessary to start observing the nest in semi-quiescence before the MFA, preferably for at least half an hour, before the massive upheaval in the nest takes place and new functions and positions are assigned to the individual bees. But this then also presupposes that one can realistically estimate the beginning of such a process, which requires some experience with giant honeybees. In the field, we succeeded in predicting the start of such a MFA with a span of one hour, taking into account comparable environmental and the general weather conditions and, in particular, the ambient temperature. Likewise, for an in-depth analysis, it is useful to observe the behavior of the experimental colony for at least one hour also after the MFA has ended*.“

I apply …

“*In this paper we investigate the question of how collective defence changes during the MFA of an Apis dorsata colony. To do this, it is necessary to start observing the nest in semi-quiescence before the MFA, preferably for at least half an hour, before the massive upheaval in the nest takes place and the individual bees are assigned new functions and positions. However, this also requires some experience with giant honey bees to be able to realistically estimate the start of such a process. In the field, we have been able to predict the onset of such an MFA with a span of one hour, taking into account comparable environmental and general weather conditions and especially the ambient temperature. It is also useful for a detailed analysis to observe the behaviour of the experimental colony for at least one hour after the end of the MFA. However, for practical reasons, the actual recording times were somewhat shorter than the actual observation time we spent at the nest.*”

# My comment:

I do not want to omit this chapter as it underlines the enormous effort, work and patience required to correctly interpret, explain and understand the behaviours associated with MFA. I have therefore also made some minor changes to eliminate the apparent contradictions with earlier statements about recording times.

******************

# Query 6

Reviewer 1: L307: even … even

# My comment:

Instead…

*“(sometimes the giant honeybees even collect nectar and pollen even at night time [60, 61])*.”

I apply…

*“(sometimes the giant honeybees collect nectar and pollen even at night time [60, 61]).”*

******************

# Query 7

Reviewer 1: L473: <1Hz rather than 0.1, if I believe the x-axis of Fig 6?

# My comment:

Instead…

“*In Fig 6A,B this motion turbulence was identified by its frequency components (Eq#4e) in the range <0.1 Hz, and was found to be distributed in a specific topographic pattern over the nest surface.*”

I apply…

“*In Fig. 6A,B, this motion turbulence was identified by its frequency components (Eq#4e) in the range from 100 Hz down to 0.1 Hz and was found to be distributed in a specific topographic pattern over the nest surface.”*

# My comment:

The diagrams concentrate on logarithmic units between 0.1 and 100 Hz, the calculations of course continue on both sides.

Done

******************

# Query 8

Reviewer 1: L493-497: sentence repeated

# My comment:

Instead...

“*In the present work, histogram-based movement spectra are used. This allows a more detailed description of how the nest evolves from the state of sQu to the state of MFA in the course of the experimental sessions, and how the MFA intensifies to its peak and then returns to sQu. As shown for episode mfa_1_ in Fig 7A, the motion spectra of the nP_d_ phases (blue lines) were monomodal and even Gaussian when the size classes were scaled logarithmically. As shown for episode mfa1 in Fig. 7A, the motion spectra of the nP_d_ phases (blue lines) were monomodal and even Gaussian when the size classes were scaled logarithmically*.”

I apply..

“*In the present work, histogram-based movement spectra are used. This allows a more detailed description of how the nest evolves from the state of sQu to the state of MFA in the course of the experimental sessions, and how the MFA intensifies to its peak and then returns to sQu. As shown for episode mfa_1_ in Fig 7A, the motion spectra of the nP_d_ phases (blue lines) were monomodal and even Gaussian when the size classes were scaled logarithmically*.”

Done

******************

# Query 9

Reviewer 1: L646-647: This sentence is weird, the grammar is wrong.

# My comment:

I don't think that the grammar is wrong. However, I have corrected this obvious inconsistency by changing the wording slightly. I admit that the first sentence of a chapter should be a bit more general.

Instead…

“*Mass flight in giant honeybees [35, 42-45, 47] manifests itself to the external observer that massive swarming behaviour takes place around such active nests, caused by triggers internal to the nest but not by any external threat.”*

I apply…

“*An attentive observer will not fail to notice that the swarming during the so-called mass flight of the giant honeybees [35, 42-45, 47] is caused by triggers within the nest and not by external threat.*”

******************

# Query 10

Reviewer 1: L652: bivouacs rather than bivaques

Instead…

“*In giant honeybees, absconding occurs regularly during migration, at comb-right nests or at bivaques [12, 13].”*

I apply…

“*In giant honeybees, absconding occurs regularly during migration, at comb-right nests or at bivouacs [12, 13].”*

Done

******************

# Query 11

Reviewer 1: L658: the surface layer of the bee curtain

# My comment:

I agree that just to avoid repetition it is fine to make this edit

Instead…

“*In giant honeybees nests, during MFA state, bees slip through the mesh of the bee curtain from the inside to the outside (see S1 Movie and description at 0.000 min), crawl around on the nest surface, fly off to circle the nest in a swarm for at least half a minute, and finally land back on the nest surface.*”

I apply…

“*In giant honeybees nests, during MFA state, bees slip through the mesh of the bee curtain from the inside to the outside (see S1 Movie and description at 0.000 min), crawl around on the nest surface, fly off to circle the nest in a swarm for at least half a minute, and finally land back on the surface layer of the bee curtain.*”

******************

# Query 12

Reviewer 1: L704: even on the other side

Instead...

„*Furthermore, there is evidence [26, 27] that with this behaviour, the colony is able to inform the nest mates about the current threat situation, even on both sides of the central honeycomb*.”

I applied..

“*Furthermore, there is evidence [26, 27] that with this behaviour, the colony is able to inform the nest mates about the current threat situation, even on the other side of the central honeycomb.*”

******************

# Query 13

Reviewer 1: L819: neither would be capable of shimmering. There and in other places, it is implied that the only defensive response available to the colony is shimmering. I think that is a bit of an over-generalization. If some bees can still detect the threat as suggested, they might still be able to individually fly out and sting/ball/harass the intruder, even if they have trouble building a strong collective response.

“*In either case, this would mean that both cohorts remain susceptible to external threats even in MFA mode, but behave in opposite ways. Neither, however, would be capable of a defensive response.*”

# My comment:

I want to leave this passage as it is. It is correct to say this in the context of MFA: “Neither, however, would be capable of a defensive response”.

We have conducted many experiments on this topic (but have not yet published them). We found that shimmering is always the first option for defence during the day, as long as the ability to shimmer has been developed since early morning and as long as the stimulation or threat intensity remains low.

When the intensity of the threat increases, for example when the visual threats in terms of geometric size and angular velocity increase, it takes a few tens of seconds to recruit a crowd of bees to fly against these visual patterns in front of the nest. When the threat level increases so immensely, this is a completely different category than when "just shimmering" is triggered, and also requires a much higher level of defence. The phenomenon of individual bees flying out of the nest to attack is obviously rare or non-existent.

Of course, there are always individual bees that catch and sting you when you are close to the nest or even further away. We experienced this not only with Apis dorsata, but also with Apis laboriosa, e.g. in Chaku, Nepal itself at a distance of 300 metres from the nest, and the great Himalayan river Bhotekoshi was even between us. However, these are always members of a small swarm that are released and search for their targets, then also singularized.

******************

# Query 14

Reviewer 1: L842: first rather than lastly

# My comment:

I would like to introduce a different idea here. I have restructured the wording accordingly.

Instead…

“*On the other hand, the ability to defend itself is in Giant honeybee nests completely suspended for a some minutes. Defensiveness lastly starts with all nest members, or at least with a strategically selected cohort, that keeps their eyes open to perceive threats coming from outside.*”

I apply…

“*On the other hand, the ability to defend itself is in Giant honeybee nests completely suspended for a some minutes. Finally, defence readiness begins when all nest members are ready and able to do so again, or at least with a strategically selected group that keeps its eyes open to detect threats from outside.*”

******************

# Query 15

Reviewer 1: L849: to an extent + to a certain degree

Instead..

“*Interestingly, the mouth zone is a kind of exception here (Fig 10A_4_,B_4_), less concerned with handling the trade-off between MFA and defensiveness. It retains to some extent its function to a certain degree as a hub for various important tasks in the nest, such as foraging, guarding, but also in this respect to trigger the signal for the beginning and end of mass flight.*”

I apply…

“*Interestingly, the mouth zone is a kind of exception here (Fig 10A_4_,B_4_), less concerned with handling the trade-off between MFA and defensiveness. To a certain extent, it retains its function as a hub for various important tasks in the nest, such as foraging, guarding, but also in this respect to signal the start and end of the mass flight*.”

Done

******************

# Query 16

Reviewer 1: Jump from Fig 4 to Fig 6, Fig 5 not used?

# My comment

In 462 (Results) Fig. 5 has already been mentioned

“*Although the motion amplitudes were normalized separately in each of the episodes (Fig 5),*…”

In fact, I have omitted to cite this Fig 5 several times. I have now made up for this (in Methods, Results and Discussion). Now I added it also in the Methods (new line 301) to have a continuation in the sequence of Figure citation; in Results (line 460), in Discussion line 715,783,791, 794 e.g. *“(Figs 4AB,5AB in contrast to Figs 4C,5C)*”

******************

# Query 17

Reviewer 1: Fig 7: red curves appear as dotted blue-red, making them hard to distinguish from the blue ones (and artboard visible in the back)

# My comment

I have widened the red line three times (to “3 mm”). The artboard is now no longer visible in the back.

******************

# Query 18

Reviewer 1: Just an idea - I feel like there could be a summary figure showing how both the MFA and defensiveness progress spatially across the nest (I mean a schematic representation, not another data-loaded figure) that would nicely wrap up the argument.

# My comment:

Thank you for this proposal. I thought about this idea for a while, but I have to admit that any suggestion I had in mind would either have been too trivial or would even have led to the wrong results. Any generalisation I tried would not, in my opinion, summarise the results satisfactorily. I'm sorry about that. Unfortunately, we have to make do with the line plots and motion spectra (and films) offered in the paper.

************

# Additionals:

I have changed some text details in the captions of the figures (such as of Fig 1, 3, 5).

************
